# Galectin-2 in Health and Diseases

**DOI:** 10.3390/ijms24010341

**Published:** 2022-12-25

**Authors:** Muhammed N. Negedu, Carrie A. Duckworth, Lu-Gang Yu

**Affiliations:** 1 Departments of Biochemistry and Systems Biology, Institute of Systems, Molecular and Integrative Biology, University of Liverpool, Liverpool L69 3GE, UK; 2 Departments of Molecular Physiology and Cell Signalling, Institute of Systems, Molecular and Integrative Biology, University of Liverpool, Liverpool L69 3GE, UK

**Keywords:** galectin-2, gastrointestinal tract, apoptosis, immune cell, pregnancy disorders

## Abstract

Galectin-2 is a prototype member of the galactoside-binding galectin family. It is predominately expressed in the gastrointestinal tract but is also detected in several other tissues such as the placenta and in the cardiovascular system. Galectin-2 expression and secretion by epithelial cells has been reported to contribute to the strength of the mucus layer, protect the integrity of epithelia. A number of studies have also suggested the involvement of galectin-2 in tissue inflammation, immune response and cell apoptosis. Alteration of galectin-2 expression occurs in inflammatory bowel disease, coronary artery diseases, rheumatoid arthritis, cancer, and pregnancy disorders and has been shown to be involved in disease pathogenesis. This review discusses our current understanding of the role and actions of galectin-2 in regulation of these pathophysiological conditions.

## 1. Introduction

Galectins are a family of 15 mammalian β-galactoside-binding proteins [1] They are chronologically numbered based largely on the order of their discoveries with the first galectin (galectin-1) discovered in 1975 from the tissues of the electric eel (*Electrophorus electricus*) [2]. Each galectin member contains one or two highly conserved carbohydrate recognition domains (CRD) of approximately 130–135 amino acids [1]. Members of the galectins are divided into three subgroups based on their structure and number of CRDs [1]. Galectins-1, -2, -5, -7, -10, -11, -13, -14, and -15 are proto-type galectins. Each proto-type galectin contains one CRD and can function as monomer (Galectin-5, -7 and -10) or homodimer (Galectin-1, -2, -11, -13, -14, and -15). Galectin-4, -6, -8, -9, and -12 are tandem repeat-type galectins, each containing two CRDs in a single polypeptide chain connected by a linker region [3,4]. Galectin-3 is the sole chimera-type galectin. It contains a single CRD at its C-terminal which is linked to a flexible N-terminal by a proline, glycine and tyrosine-rich collagen-like sequence [4].

Galectin-2 is a proto-type galectin and was first identified in 1992 as a coding sequence in a cDNA library of human HepG2 hepatoma and was later cloned and isolated from HepG2 cells [5]. Galectin-2 can form homodimers which provides it with the ability to crosslink binding receptors on the cell surface [6]. Galectin-2 is predominately expressed in the gastrointestinal tract but is also abundant in other tissues such as the placenta [7] and in the cardiovascular system [8,9]. Galectin-2 has been shown to be involved in the regulation of several physiological and pathological conditions such as epithelial layer integrity, inflammation, immune response and apoptosis [10,11]. Altered expression of galectin-2 is observed in inflammatory bowel disease, several pregnancy-related disorders and various cancers and has been shown to be involved in their disease pathogeneses [12,13,14].

## 2. Galectin-2 Structure

**Figure 1:**
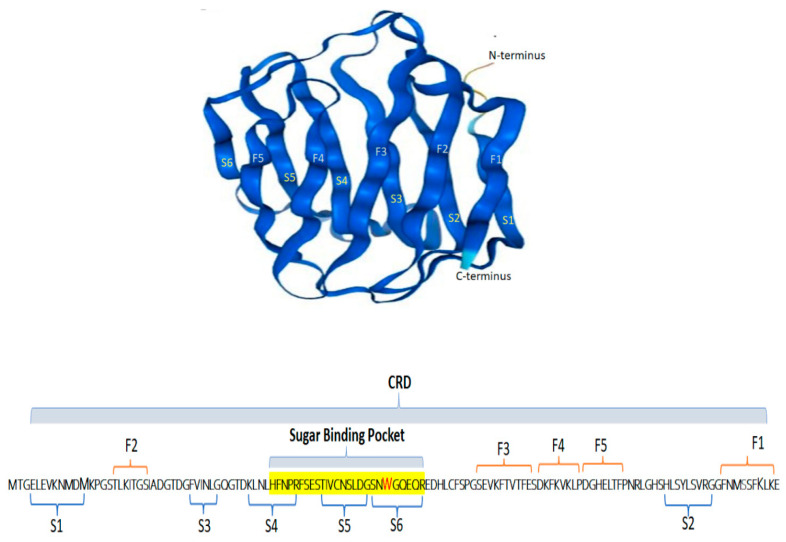
The structure of human galectin-2. The **top** panel shows galectin-2 molecule in 3D (Protein Atlas HGNC:6562) with the two beta sheets S1–S6 and F1–F5 marked. The **bottom** panel shows the galectin-2 amino acid sequence with mapped regions of the two beta sheets and the sugar binding pocket (with the tryptophan Trp65 residue in red).

The galectin-2 gene *LGALS2* in human is located on chromosome 22. Its location is very close to the galectin-1 gene *LGALS1* (50 kbp apart) which is on the opposite strand of the same chromosome [15]. Galectin-2 is a 14 KDa protein with one CRD at the C-terminal [5,16,17]. The galectin-2 CRD is divided into subsites A to D [18]. Subsite C is the defining galactoside-binding site and contains six of the seven highly conserved amino residues of the CRD [19,20]. Subsites A, B and D contribute to the binding of galectin-2 with saccharides flanking the β-galactoside residue [18,20]. Galectin-2 has a β-sandwich structure formed by two β-sheets with 5- (F1–F5) and 6- (S1–S6) β-strands on each side of CRD [16] (Figure 1). Galectin-2 exists as a monomer but is capable of forming non-covalent homodimers in solution [17]. Galectin-2 dimerises on β-strand F1 and S1, which is opposite to the sugar binding site S4–S6, in each monomeric unit [21]. The dimer interface of galectin-2 is formed by four parallel hydrogen bonds in each β-sheet with contribution of residues 4–10 and 125–131 from each monomer [16]. An early study showed that both monomer units within the galectin-2 dimer are involved in ligand binding [16] while a more recent study suggested the involvement of possibly only one monomeric unit [17]. Like other proto-type galectins, galectin-2 harbours a tryptophan residue (Trp65) in its CRD and the change of tryptophan fluorescence in response to galectin-2 ligand binding can be used to determine the strength of galectin-2-ligand interactions [20].

Like other galectin members, galectin-2 is synthesized in the cytosol in mammalian cells and can be transported to the cell nucleus, cell membrane or outside cells after synthesis [22,23]. Galectin-2 translocation into the nucleus could be through a similar pathway as widely studied galectin-3 either by passive diffusion or active transportation by partnering with nuclear transport proteins [24,25]. Like other galectin family members, galectin-2 does not contain a secretion signal sequence and its secretion to outside cells is believed through non-classical pathways and probably involves endosomes [26].

## 3. Galectin-2 Binding Ligands

Galectin-2 binding to galactose-terminated complex oligosaccharides is stronger than its binding to galactose monosaccharide or galactose-containing disaccharides [27]. For example, galectin-2 binding to N-acetyl-lactosamine (LacNAc) is over 50-times stronger than its binding to galactose [20] (Table 1). 3-O-sulphation of the galactose residue in LacNAc also enhances galectin-2 binding [27,28]. A number of cellular glycoproteins have been reported to be bound by galectin-2. These include β_1_ integrin on human T cells [29] ganglioside GM_1_ in neuroblastoma cells [30], MUC1 in epithelial cancer cells [12,31] and MUC5AC on gastric mucous [6]. Lymphotoxin-α and β-tubulin have also been reported to interact with galectin-2 in macrophages and in atherosclerotic plaque of the heart muscle following myocardial infarction [32]. Galectin-2 can bind the glycans on A, B and O blood group antigens that carry fucose- and galactose-modified LacNAc structures [33]. Binding of galectin-2 to these blood group-related glycans is crucial in galectin-2-mediated agglutination of erythrocytes [34].

## 4. Galectin-2 in the Digestive System

Galectin-2 is predominantly expressed by gastrointestinal epithelial cells, especially the mucous neck cells and surface mucous cells of the stomach and goblet cells in the small intestine, brush border of the intestinal enterocyte and colon [36,37,38,39]. Galectin-2 secretion by mouse gastric epithelial cells has been shown to bind to mucin protein MUC5AC and crosslink mucin proteins in the mucous [6]. This was shown to contribute to the strength of the mouse mucus layer and enhances its ability to protect the epithelium [40]. Galectin-2 has also been reported to bind β-catenin on epithelial cell membrane resulting in enhancing the complex formation between β-catenin and cytosolic E-cadherin. This interaction was shown to enhance adhesion and migration of human colon cancer caco-2 and non-transformed rat intestinal epithelial IEC-6 cells [14]. Galectin-2-β-catenin complex formation also showed to promote gastrointestinal wound healing and barrier strength during chronic and acute DSS induced and adoptive transfer colitis in mice [14,41]. No difference in galectin-2 concentration was however seen in the blood circulation between healthy and ulcerative colitis and Crohn’s disease patients [42].

**Figure 2:**
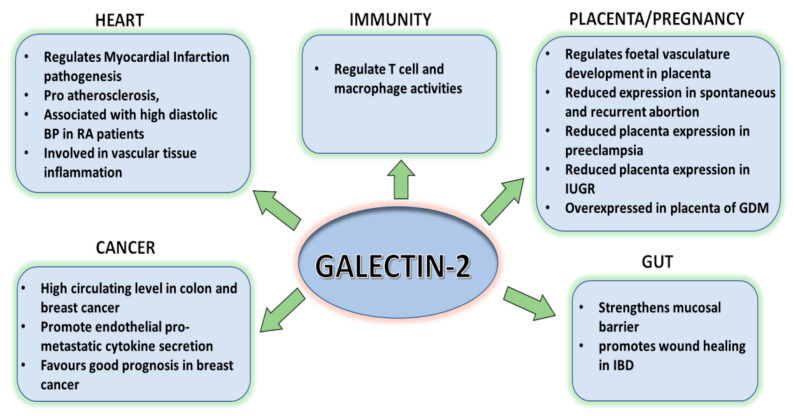
Putative effect of galectin-2 in pathophysiology. Studies in mice have suggested that galectin-2 expression and secretion in the gastrointestinal tract contribute to mucosal barrier maintenance and preserve epithelial architecture. Galectin-2 expression in the placenta has shown to support foetal vasculature development in pregnancy. Galectin-2 has also been reported to be involved in MI pathogenesis and atherosclerosis in the heart. Galectin-2 expression in breast cancer was shown to correlate with good prognosis. Higher level of circulating galectin-2 showed to promote tumour cell hematogenous dissemination and endothelial secretion of metastasis-promoting cytokines.

In an animal study, galectin-2 expression was observed to be substantially lower in the colonic lamina propria of DSS-treated colitis mice in comparison to untreated control mice [43]. Administration of exogenous galectin-2 at the onset of DSS-induced colitis preserved the integrity of colonic crypts and epithelial architecture that would otherwise be caused by DSS damage [43]. Galectin-2 administration also showed to increase the release of anti-inflammatory cytokine IL-10 and decrease the release of pro-inflammatory cytokines IL-6 and IL-12p70 by intestinal and lamina propria-associated mononuclear cells [43]. Mice administrated with exogenous galectin-2 displayed higher amounts of T cell apoptosis in the lamina propria area in comparison to control animals [43]. All these studies indicate that galectin-2 expression in the GI tract may contribute to maintaining the mucosal barrier by protecting epithelial integrity (Figure 2). It should be pointed out however that all these in vivo studies described above were conducted in mice. Whether similar effect of galectin-2 also occurs in human still remains to be determined.

## 5. Galetin-2 in Pregnancy

Expression of galectin-2 is detected in the syncytiotrophoblast (STB) and extravillous trophoblast (EVT) of the normal placenta [44]. Overexpression of galectin-2 was observed in foetal STB and maternal decidua of gestational diabetes mellitus (GDM) placenta [45]. Higher amounts of galectin-2 expression in foetal syncytiotrophoblast and maternal decidua cells in GDM showed to reduce the development of foetal vasculature by stimulating macrophage secretion of pro-inflammatory cytokines in the decidua [45]. Analysis of spontaneous and recurrent abortion cases in early stages of pregnancy revealed a significant reduction of galectin-2 expression in villous and EVT at the foeto-maternal interface in comparison to cases of induced abortion [46]. Interestingly, reduction of galectin-2 expression was observed in the placenta of all compartments of male, but not female, intrauterine growth restriction (IUGR) cases in the third trimester of pregnancy [47]. It has been speculated that reduction of galectin-2 expression in the placenta may lead to overactivation of the immune response to maternal tissue or to failure of foetal implantation as a result of reduced T cell apoptosis [46].

Preeclampsia (PE) is a pregnancy-associated disorder characterised by hypertension and proteinuria [48]. Galectin-2 expression has been reported to be two-fold lower in the decidua of PE patients than in that of normal pregnancy [49]. The reduction of placental galectin-2 expression in the decidua, STB and EVT was seen to be associated with PE pathogenesis [7]. It is not yet known whether the reduction of galectin-2 expression influences the development of IUGR or whether galectin-2 reduction is a consequence of failed trophoblast invasion [47]. On the other hand, the presence of galectin-2 in the placenta of patients with preeclampsia has been reported to reduce regulatory T cell (FoxP3^+^) apoptosis [13]. As apoptosis of regulatory T cells is a major contributor to immunity-related pregnancy disorders [46], galectin-2-mediated reduction of T cell apoptosis may help to prevent immunity related pregnancy disorders [13]. Overall, all the studies so far point to a beneficial role of galectin-2 expression during pregnancy (Figure 2). However, most of the human studies in this area included only limited patient numbers and further studies with larger sample size are still needed to confirm the discoveries from these studies.

## 6. Galectin-2 in Immunity

A number of studies have reported the involvement of galectin-2 in the regulation of immune response and T cell activation [50]. Although galectin-2 is not detected in T cells [29], its presence can activate T cells [51] and induce T cell apoptosis [13] by binding to cell surface glycoproteins such as CD3 and CD7 on T cells. Binding of galectin-2 to activated T cells has been shown to enhance the secretion of IL-5, IL-10 and decrease the secretion of IFN-γ and TNF-α by T cells [29]. The presence of galectin-2 shows to favour the conversion of activated T cells to T helper cells in myocardial infarction [50,51]. Introduction of galectin-2 to monocytes in culture prevented *Salmonella* induced upregulation of MHC class II molecules and enhanced monocyte secretion of TNF-α and IL-10 resulting in cell apoptosis [52]. Induction of cell apoptosis by galectin-2 has also been reported to occur in neutrophils in vitro [53]. Although the precise mechanisms of galectin-2 involvement in the regulation of immune response still remain largely unknown, its induction of immune cell apoptosis may represent one of the main actions. It should be mentioned that the immunomodulatory activity of galectin-2 shown in these studies is likely involved in galectin-2-mediated regulation of disease pathogenesis such as in pregnancy-associated disorders discussed early and in cardiovascular dis-orders to be discussed below. This is an area that clearly warrants good attention in future studies.

## 7. Galectin-2 in the Cardiovascular System

The involvement of galectin-2 in cardiovascular events was first implicated from the identification of galectin-2 as a binding partner of lymphotoxin-α, a cytokine that is involved in the pathogenesis of myocardial infarction (MI), in COS7 non-steroidogenic and U937 pro-monocytic cells [32]. Subsequent investigations have revealed the existence of 17 single nucleotide polymorphisms (SNPs) of galectin-2 gene in the coronary arteries of MI patients [32]. The presence of one of the SNPs, SNP 3279C-T in intron 1, showed to correlate with MI pathogenesis of elderly patients in the Japanese population [32,54]. However, such an association between galectin-2 SNP in intron 1 and MI was not seen in German and British populations [55], indicating possible different frequencies and patterns of galectin-2 SNP between Caucasian and Japanese populations [55,56,57,58].

Three different genotype groups of galectin-2 SNPs (CC, CT and TT genotypes) in intron 1 have been reported to be associated with high diastolic blood pressure in patients with rheumatoid arthritis [59]. The presence of galectin-2 SNP in intron 1 (3279C/T) was suggested to be a marker of high risk of cardiovascular events in rheumatoid arthritis [59]. Colocalization of galectin-2 with BRCA1-associated protein (BRAP) was observed in the cytoplasm of coronary artery smooth muscles and macrophages [60]. Galectin-2 gene polymorphism, together with *CXCL16*, *AGTR1* and *PPARG* gene polymorphism, was shown to be closely associated with the development of coronary heart diseases in patients [61]. How galectin-2 polymorphism is involved in coronary heart disease development is however unknown. As galectin-2 has been reported to promote secretion of lymphotoxin-α, an inflammatory cytokine from macrophage cells [32], its influence on tissue inflammation may be a possible contributor to its action.

In animal studies, regular intraperitoneal injection of galectin-2 showed to markedly impair perfusion restoration, following left femoral artery coagulation, and reduce mean arterial lumen and macrophage population around the coagulated artery in a murine hindlimb model [8,9]. Conversely, administration of an anti-galectin-2 antibody increased perfusion restoration, mean arteriolar diameter and the number of M2 macrophages around the artery [62]. Administration of anti-galectin-2 antibody also inhibited the progression of atherosclerosis and favoured the generation of new arteries in ischemic heart disease in a mouse model [63]. Together, these studies suggest that regulation of tissue inflammation by galectin-2 may represent an important mechanism in the pathogenesis of cardiovascular disorders.

## 8. GALECTIN-2 in Cancer

**Figure 3:**
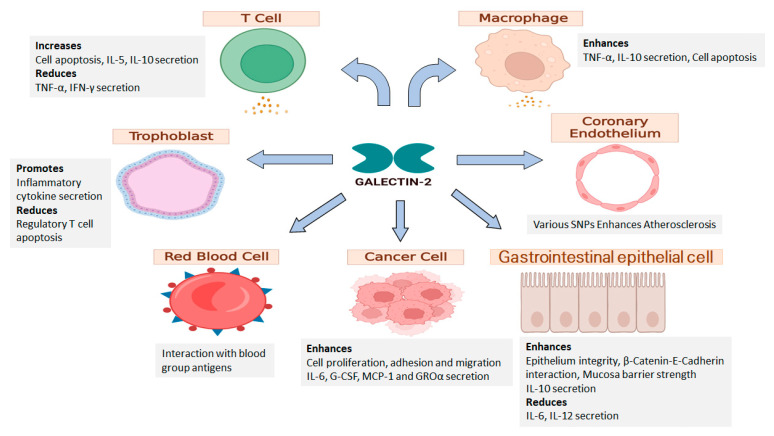
Possible effects of galectin-2 on activity of different cell types. Studies using mouse gastric epithelial cells have suggested the involvement of galectin-2 in protection of epithelial integrity of the GI tract. Several studies have also reported a role of galectin-2 in promoting immune cell apoptosis and induction of cytokine secretion by T cells, macrophages, trophoblasts and cancer cells. Galectin-2 was shown to recognize the blood group-associated glycans and to contribute to erythrocyte agglutination. Multiple galectin-2 SNPs are observed in cardiovascular and have been proposed to regulate tissue inflammation. Reduction of galectin-2 expression by trophoblasts has shown to be associated with pregnancy-associated disorders.

Several studies have reported an association of galectin-2 expression with cancer prognosis in breast and colon cancer patients. High level of galectin-2 mRNA was shown to correlate with a favourable prognosis and overall survival in HER-2 overexpressing breast cancer and in luminal B-type breast cancer patients [64]. A multidimensional CRISPR screening study in mice on the other hand suggested that the galectin-2 gene is a positive regulator of immune escape in triple-negative breast cancer [65]. Tumours inoculated with galectin-2 transfected triple-negative breast cancer 4T1 cells in BALB/c mice showed to grow significantly faster and bigger than inoculated with control vector transfected 4T1 cells and administration of an anti-galectin-2 antibody reduced the tumour growth [65]. A reduced level of galectin-2 was observed in lesion of *Helicobacter-*induced gastric cancer in mice in comparison to normal mice [66]. A 12 fold higher level of galectin-2 was observed in lymph node metastasis-negative gastric cancer tissue than in advanced and lymph node metastasis-positive gastric cancer tissue [67]. This indicates that high galectin-2 expression within the tumour may be associated with a favourable outcome for gastric cancer patients. Interestingly, levels of galectin-2 in the blood circulation have been shown to be significantly higher in both colon and breast cancer patients in comparison to healthy people [31]. Patients with metastasis were shown to have even higher amounts of circulating galectin-2 than those with only localised tumours [31]. Binding of galectin-2 to the oncofetal Thomsen-Friedenreich antigen Galβ1,3GalNAcα- (TF antigen) on the mucin protein MUC1 of tumour cells enhanced cancer cell adhesion to the vascular endothelium [12]. Circulating galectin-2 was also shown to interact with vascular endothelial cells which induced endothelial secretion of metastasis-promoting cytokines G-CSF, IL-6, GROα and MCP-1 [68] (Figure 3). These studies indicate that galectin-2 expression at different locations like serum and gastrointestinal mucosa may have very different biological influence on different cancer types. Although the molecular mechanisms behind these differences are unknown, expression of different galectin-2 binding glycans by different cancer types may be an important contributor to these different galectin-2 actions.

## 9. Concluding Remarks

Compared to the more widely expressed galectin members galectin-1 and galectin-3, galectin-2 expression in the body is relatively more restricted. It is predominately expressed in the gastrointestinal tract although is also detected in other tissues such as the placenta and cardiac vasculature. studies so far suggest that galectin-2 expression and secretion may contribute to enhancing the mucosal barrier of the gastrointestinal tract and be involved in regulation of the pathogenesis of coronary artery diseases, rheumatoid arthritis, cancer, and pregnancy disorders. However, the true nature and importance of galectin-2 actions in homeostasis and pathophysiology remain largely unknown. More studies are needed to understand the precise molecular mechanisms of galectin-2 involvement in these pathophysiological conditions, including the identities of the galectin-2 binding glycans/receptors. As galactose-terminated glycans are expressed by many cell membrane glycoproteins which could be recognized by galectin-2, future research may reveal opportunities for therapeutic intervention of galectin-2-associated health issues.

## Figures and Tables

**Table 1:** Binding of galectin-2 to galactose-terminated glycans. * TFS, Tryptophan Fluorescence Spectroscopy; ITC, Isothermal Titration Calorimetry; FAC, Frontal Affinity Chromatography.

Glycans	K_D_ Value	Assesment Methods	Reference
Galactose	35.6 mM	TFS *	[20]
Lactose (Galβ1-4GlcNAc)	1.3 mM		
*N*-acetyllactosamine (Galβ1-4GlcNAc, LacNAc)	654.4 µM		
Lactose	960.5 µM	ITC	
*N*-acetyllactosamine	554.2 µM		
Galβ1-3GlcNAc	68 µM	FAC	[35]
*N*-acetyllactosamine	130 µM		
GM1	240 µM		
Galβ1-4GlcNAcβ1-3Galβ1-4GlcNAc	140 µM		
Galβ1-4GlcNAcβ1-3Galβ1-4GlcNAcβ1-3Galβ1-4GlcNAc	90 µM		
Galβ1-4GlcNAcβ1-3Galβ1-4GlcNAcβ1-3Galβ1-4GlcNAcβ1-3Galβ1-4GlcNAcβ1-3Galβ1-4GlcNAc	85 µM		

## Abbreviations

ACT1, Angiotensin II Receptor Type 1; AGTR1, Angiotensin II receptor type 1; BRAP, BRCA-1 Associated Protein; BRCA-1, Breast Cancer Gene 1; CHD, Coronary Heart Disease; CRD, Carbohydrate Recognition Domain; CXCL16, Chemokine ligand 16; DSS, Dextran Sulphate Sodium; FAC, Frontal Affinity Chromatography; G-CSF, Granulocyte Colony Stimulating Factor; GDM, Gestational Diabetes Mellitus; EVT, Exravillous Trophoblast; GI, Gastrointestinal; GROα, Growth Regulated Oncogene α; HER2, Human Epidermal Growth Factor Receptor 2; IBD, Inflammatory Bowel Disease; IFN-γ, Interferon-γ; IL-, Interleukin; ITC, Isothermal Titration Calorimetry; IUGR, Intrauterine Growth Restriction; LacNAc, N-acetyllactosamine; MCP-1, Monocyte chemoattractant Protein-1; MHC, Major Histocompatibility Complex; MI, Myocardial Infarction; PE, Preeclampsia; PPARG. Peroxisome Proliferator Activated Receptor Gamma; SNP, Single Nucleotide Polymorphism; STB, Syncytiotrophoblast; TF antigen, Thomsen Friedenreich antigen galactoseβ1,3N-Acetyl-galactosamineα-Ser/Thr-4; TFS, Tryptophan Fluorescence Spectroscopy; TNF-α, Tumour Necrosis Factor-α.
